# An evaluation of the global network of field epidemiology and laboratory training programmes: a resource for improving public health capacity and increasing the number of public health professionals worldwide

**DOI:** 10.1186/1478-4491-11-45

**Published:** 2013-09-21

**Authors:** Renee E Subramanian, Dionisio G Herrera, Paul M Kelly

**Affiliations:** 1 The Task Force for Global Health Inc, TEPHINET Program, 325 Swanton Way, Decatur, Georgia 30030, USA; 2 ACT Government Health Directorate, Australia and Australian National University Medical School, GPO Box 825, Canberra City, Australian Capital Territory (ACT) 2601, Australia

**Keywords:** Epidemiology, Public health, Training, Global, TEPHINET, FETP, FE(L)TP, Capacity building, Regional networks, EIS

## Abstract

**Background:**

Given that many infectious diseases spread rapidly, across borders and species, there is a growing worldwide need to increase the number of public health professionals skilled in controlling infectious epidemics. Needed also are more public health professionals skilled in non-communicable disease surveillance and interventions. As a result, we surveyed all 57 field epidemiology training programmes (FETPs) that are members of the Training Program in Epidemiology and Public Health Interventions Network (TEPHINET), to evaluate the progress of the FETPs, the only global applied epidemiology network, toward increasing public health capacity globally.

**Methods:**

Data on the FETP programmes and the training they provide were abstracted from TEPHINET membership surveys and verified with FETP directors for all FETPs that were members of TEPHINET in 2012. Data on abstracts submitted to the recent TEPHINET Global Scientific Conference, on recent accomplishments by each FETP, and on quality improvement were also compiled to provide a worldwide view of the public health human resource capacity produced by these programmes.

**Results:**

A total of 6980 public health professionals worldwide have graduated from an FETP or from the Center for Disease Control and Prevention’s Epidemiology Intelligence Service (EIS). FETP residents and graduates participate in key public health prevention, control, and response activities. Each FETP has adapted its curriculum and objectives over time to align with its country’s public health priorities. FETPs are well integrated into their national public health infrastructures, and they have many partners at the national, regional and global levels.

**Conclusion:**

FETPs are a competent and diverse source of highly skilled public health professionals who contribute significantly to public health’s global human resource needs. This finding is evidenced by 1) the training curricula that were adapted over time to meet public health’s human resource needs, 2) the FETPs’ continued support from internal and external partners, 3) the increasing number of FETP residents and graduates and their increasing contribution to effective public health work, and 4) the increased quality improvement initiatives facilitated through the FETPs membership in one global network, TEPHINET.

## Background

The number and calibre of public health professionals skilled in preventing and responding to disease epidemics needs to increase to combat the growing number of endemic, emerging and re-emerging infectious diseases that spread rapidly across borders, through countries and eventually throughout the world [1]. It is the mission of the field epidemiology training programmes (FETPs) to teach these skills, which now exist in 57 countries, up from 10 countries in 1997. The number of countries that send health professionals for training to FETPs has also grown from 10 to 84 [2]. All 57 FETPs belong to one global network, the Training Program in Epidemiology and Public Health Interventions Network (TEPHINET), which is headquartered in Atlanta, Georgia, with branch offices in Pakistan, Colombia, and Guatemala. TEPHINET’s mission is to strengthen global public health capacity by supporting well-qualified professionals in field epidemiology through training, service and networking opportunities. TEPHINET is the only global network of applied epidemiologists. To evaluate how well its worldwide network of FETPs is fulfilling the mission to increase public health capacity, TEPHINET compiled data from all 57 FETPs that were part of the global network in 2012, to review common elements of the training curricula, key activities and achievements of the FETP residents and graduates, including the ability to share best practice at scientific conferences, and activities of the global network to improve the quality of training.

### The FETP focus on learning through service

Most FETPs are modelled on the Epidemic Intelligence Service (EIS), established in 1951 at the Centers for Disease Control and Prevention (CDC) or the Rockefeller Foundation Public Health Schools Without Walls model [3,4]. In contrast to other public health training programmes, FETPs often rely on graduates to mentor first-year residents [3]. FETPs also require residents to support the host institution priorities through field assignments during which the residents apply the concepts learned during didactic teaching. All FETPs share this common value of learning through service. Graduates often move into influential positions within their country’s Ministry of Health (MOH) or within a local or international public health organization, thereby enhancing the country’s evidence-based, public health policy and practice. Throughout the history of FETPs, residents and graduates have been key responders to some of the highest priority public-health challenges around the world, including epidemics related to HIV/AIDS, severe acute respiratory syndrome (SARS), West Nile virus, and avian (H5N1) and swine (H1N1) influenza. Graduates and residents also responded to natural disasters such as the 2004 Indian Ocean tsunami and the 2010 earthquake in Haiti [5-7].

FETPs are typically hosted by a country’s MOH or national public health institute and are closely affiliated with local public health institutions and universities. The institutions that host FETPs are vitally important to their development and sustainability because they provide space, equipment, staff, expertise and a potential career path for residents.

### FETP regional programmes and networks

During the past 5 to 7 years, regional networks of FETPs have formed to increase the capacity of the global network to share information and implement quality improvement measures. The regional networks comprise several countries in the same geographic area. Some regional FETPs have headquarters in one country and train residents of neighbouring countries: examples are the European Programme for Intervention Epidemiology Training in Sweden and the Central American FETP based in Guatemala. The FETP regional networks include the Eastern Mediterranean Public Health Network and the Southeast Asian Field Epidemiology Training Network, both established in 2009, and the African Field Epidemiology Network established in 2005. These regional networks are registered as local non-profit entities in Jordan, the Philippines and Uganda, respectively. Although each region of FETPs is unique in its development and governance, all regional networks collaborate with TEPHINET and all share the same mission: to increase public health capacity by supporting strong, effective and sustainable applied epidemiology training programmes for health professionals [8,9].

### Partners in implementation

Most FETPs received technical support or funding for implementation from CDC. Others received support from their local government, local public health institution or a partner, such as the World Health Organization (WHO). Currently CDC provides the majority of technical support to FETPs, however, numerous collaborators such as the WHO, foundations, and private and non-profit organizations also provide significant contributions to FETP activities.

## Methods

This evaluation included an analysis of membership data and recent accomplishments from all FETPs that were part of the global network in 2012, descriptive data on abstracts submitted by FETP residents and graduates to the TEPHINET Global Scientific Conference in 2012 and a qualitative analysis of efforts to increase the quality of training.

In order to obtain membership to TEPHINET, each FETP submits responses, either online or by Microsoft Excel, to a membership survey that focuses on general programme information, the residents and graduates of the programme, collaborating organizations and characteristics of the training. The TEPHINET board of directors developed the membership survey in 2006, and only a few minor modifications were made thereafter to increase its acceptance by the members and to ensure that longitudinal data were comparable. FETPs are requested to update this information every 2 to 3 years.

Data on FETP course offerings, the training competencies (that is, outbreak investigation, surveillance system development and evaluation and management and leadership, et cetera), and criteria for residents to apply to the programme, the proportion of field versus didactic training, and collaborating agencies and organizations were abstracted from the membership surveys. Cumulative data that may increase or fluctuate each year, such as the number of graduates or activities completed by residents, had been updated between August and November of 2012 for all programmes. Key activities and accomplishments of programmes in 2012 were added to the repository of programme information. FETPs were contacted by e-mail about missing or unclear data. All final data were cross-checked for accuracy with previously collected data and directly verified by conference call with FETP directors.

Descriptive data were obtained from a database of all abstracts submitted to the November 2012 TEPHINET global scientific conference, to identify current activities and accomplishments of the programmes and residents. TEPHINET conferences are a primary forum for residents and graduates to present their work to an international audience of experts and gain valuable feedback; they also serve as an opportunity for public health partners to learn about FETP activities.

Information about quality improvement efforts were obtained from globally representative scientific committees that regularly convened between July 2010 and December 2012 and from the July 2009, 2010, and November 2012 programme directors’ meetings. These efforts built on the *Continuous Quality Improvement* manual developed in 2005 to standardize field epidemiology training and increase quality, and launched a process for FETPs to voluntarily become accredited in field epidemiology training by an independent accrediting body [10,11]. The purpose of accreditation is not only to improve the quality of the FETP, but to assure partners about the capacity of residents and graduates to tackle challenging public health issues.

## Results

### FETP curricula

All FETP training programmes value learning by doing or learning through service, and all report that at least 60% of training is in-service and supports national public health priorities. On average, 76% of the training is completed in the field, and only 24% is didactic. However, the proportion of time spent in field activities ranges from 60% to 90%. In part, this variation is due to the didactic requirements for a master’s degree, because 58.5% of FETPs now grant a Master of Public Health (MPH) or a Master of Epidemiology. The role of an FETP within an MOH or country’s public health structure also affects the proportion of field versus didactic training offered.

In addition to a master’s degree, many FETPs now offer specializations in laboratory science (thirteen FETPs) or veterinary epidemiology (seven FETPs). The veterinary specialization was established recently to address the infectious zoonotic diseases that are endemic, emerging, or re-emerging. Other specializations, offered by four FETPs, include public health programme management, monitoring and evaluation, social and public health sciences, health inspection and health education.

**Figure 1:**
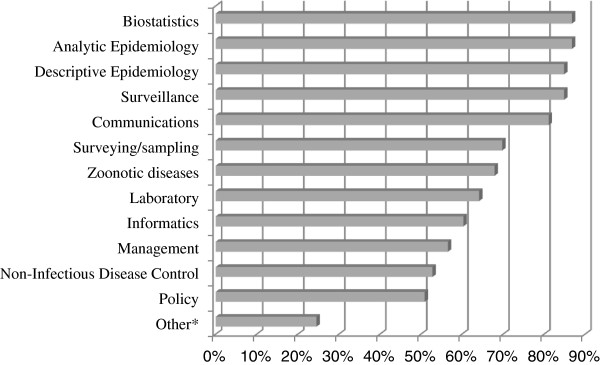
Percentage of field epidemiology training programmes (FETPs) that offer training in each of 13 subjects. ^*^Other includes bioethics, health economics, scientific writing, software for data analysis, research methods, programme evaluation, health promotion, qualitative research, media relations, laboratory diagnostics, demography, health systems management, reproductive health, assessing emergency situations, injury prevention and occupational health.

The top five courses offered by over 80% of FETPs (Figure 1) align with the core competencies outlined in the *Continuous Quality Improvement* manual. These courses are in biostatistics, analytical epidemiology, descriptive epidemiology, surveillance and communications. Courses offered by fewer than half of FETPs are in bio-ethics, health economics, scientific writing, research methods, leadership, programme evaluation, health promotion and education, conflict management, media relations, laboratory diagnosis, demography, health systems management, reproductive health, time series analysis, rapid assessment of emergency situations, vaccine preventable diseases, injury prevention, and environmental and occupational health.

### Collaborating organizations and agencies

Most (41 of 57) FETPs report being hosted by one public health institution: of these, 21 were hosted by an MOH, 15 by a public health institute and 5 by a local university. The remaining 16 FETPs are hosted by two institutions (Table 1). Other institutions and organizations that programmes collaborate with at the national or regional level, mainly for surveillance and response activities, are ministries of agriculture, livestock organizations, health services academies, national centres for communicable disease, and the US Naval Medical Research Detachment.

**Table 1:** Percentage of FETPs by type of host institution

**Host institution**	**FETPs, number**	**FETPs, percent**
Ministry of health	21	36.84%
Public health institute	15	26.32%
Ministry of health and local university	11	19.30%
Local university	5	8.77%
Public health institute and local university	3	5.26%
Ministry of health and public health institute	2	3.51%
**Total**	57	100.00%

*FETP* field epidemiology training programme.

International public health organizations that collaborate with FETPs for surveillance and response are the CDC, European Centre for Disease Prevention and Control, WHO, Food and Agricultural Organization of the United Nations, World Organization for Animal Health, US Agency for International Development, US Food and Drug Administration, US Department of Defense and United Nations Children’s Funds.

### FETP residents and graduates

Admission to an FETP is highly competitive. Selection criteria include educational qualifications, such as a medical or doctoral degree in a related field, credentials, work experience, current position in public health and epidemiologic skills. Other criteria that may also be considered are motivation, language skills, communication and computer skills, personality, leadership abilities, expectations of the programme and availability to work in the geographic area of interest.

Since the inception of TEPHINET in 1997, the global network of FETPs (excluding the US EIS programme) collectively graduated 3678 epidemiologists, which is approximately 73% of all those who enrol. The epidemiology training programme on which most others are modelled, the EIS, has graduated 3302 graduates since 1951, which means a total of 6980 field epidemiologists trained since 1951 (Table 2). The number of residents currently enrolled in an FETP somewhere in the world is 1269. Before beginning an FETP training programme, most graduates are physicians (51%), with the remainder being epidemiologists (22%), veterinarians (9%), laboratory specialists (9%), pharmacists (3%) or some other type of health professional (6%).

**Table 2:** Number of residents and graduates of a field epidemiology training programme^* ^**as of August 2012**

**World Health Organization region**	**Africa**	**Americas**	**Europe**	**Eastern Mediterranean**	**Southeast Asia**	**Western Pacific**	**Total**
Residents enrolled in 2012	290	379^*^	183	101	105	218	1108
Graduates since FETP first offered	574	4101^*^	546	291	310	1158	6980
Countries with an FETP or FE(L)TP	Ethiopia	Argentina	Central Asia^†^	Egypt	India-Chennai	Australia	
	Ghana	Brazil	EPIET	Iraq	India-Delhi	China	
	Kenya	Canada	France	Jordan	Indonesia	Hong Kong	
	Mozambique	Colombia	Germany	Morocco	Thailand	Japan	
	Nigeria	Costa Rica	Italy	Pakistan		Laos	
	Rwanda	Dominican Republic	South Caucasus	Saudi Arabia		Malaysia	
	South Africa	El Salvador	Spain	Yemen		Mongolia	
	Tanzania	Guatemala	United Kingdom			Philippines	
	Uganda	Honduras				Singapore	
	West Africa	Mexico				South Korea	
	Zimbabwe	Nicaragua				Taiwan	
		Panama				Vietnam	
		Paraguay					
		Peru					
		United States (EIS)					

^*^These figures include 161 enrolled residents and 3302 graduates of the Center for Disease Control and Prevention’s EIS, which started in 1951 and is the model for most FETPs and FE(L)TPs. ^†^Includes Kazakhstan, Kyrgystan, Tajikistan, Turkmenistan and Uzbekistan.

*FETP* Field Epidemiology Training Programme, *FE(L)TP* Field Epidemiology and Laboratory Training Programme, *EIS* Epidemiology Intelligence Service, *EPIET* European Programme for Intervention Epidemiology Training.

A snapshot of the career path of graduates from one to two countries from each region shows that about 50% of the graduates reported working in their country’s MOH and close to 70% reported working in a governmental organization (including the MOH) for 5 years or more following graduation. In the Dominican Republic, for instance, all managerial epidemiology positions for the WHO International Health Regulations (IHR) are held by graduates of the Dominican Republic’s FETP. The 2009 to 2012 data from the Central Asia Regional FETP showed that 100% of its graduates who worked in the region had positions in the government’s health system.

### Some recent FETP activities and accomplishments

**Figure 2:**
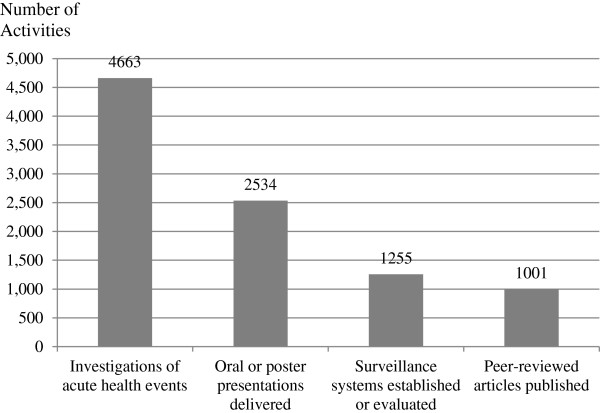
Key activities and accomplishments by field epidemiology training programme (FETP) residents (2009 to 2012).

Residents’ accomplishments indicate that FETPs can strengthen a country’s public health capacity. Examples of residents’ accomplishments are the number of peer-reviewed papers published presentations delivered at a national, regional or global scientific conference; surveillance systems developed or evaluated and disease outbreaks investigated (Figure 2). For instance, Ethiopia’s FETP was established in 2011, and as of August 2012, its residents had conducted 44 outbreak investigations and participated in developing or evaluating more than 20 surveillance systems. Ghana’s FETP was involved in several different types of outbreaks, including yellow fever, diarrhea, measles, cholera and rabies.

In the Americas, 49 EIS officers either deployed to Haiti or served in the Emergency Operations Center at CDC after the 2010 earthquake. In the Middle East, Pakistan’s FETP collaborated with the WHO and CDC to identify 53 high-risk areas for the project - Stop the Transmission of Polio, and 5360 children who needed to be vaccinated. In Asia, China and India both reported the addition of chronic disease training to their programmes.

In addition to these outputs, residents and graduates of the programmes submitted 759 abstracts from 52 countries to the most recent TEPHINET global scientific conference in Amman, Jordan in November 2012. This was the largest submission of abstracts to a TEPHINET scientific conference. Out of the 759 abstracts submitted 231 were selected for oral or poster presentation. Presentation topics included HIV, influenza (H1N1), malaria, cholera, tuberculosis, measles, nutrition, road traffic injuries, brucellosis and vaccine-preventable diseases. The selected abstracts showed the impact of FETPs through their descriptions of 1) the application of epidemiology techniques in the field and 2) the practical interventions that occurred as a result of analysing data gathered during field investigations.

One example at the conference of the work completed by residents in the field was a case–control study in Uganda on risk factors associated with road traffic injuries, which account for 44% of all injuries in Uganda. Specifically the study looked into the portion of injuries that were motorcycle-related and found that using headlights during the day, having a post-primary education, and having at least 5 years’ experience riding a motorcycle protected against motorcycle-related injuries.

Another abstract focused on a 2012 investigation into an observed increase in the number of cases of, and deaths from, severe acute respiratory infection in Brazil. The cases were confirmed to be influenza Type A (H1N1). Investigators concluded that late diagnosis and treatment in addition to a previous diagnosis of heart disease explained the increase. As a result, investigators recommended that health professionals diagnose and treat respiratory infections more aggressively and that influenza vaccination programmes be expanded to include more people, particularly those with co-morbidities.

### Quality improvement

Quality improvement for epidemiology field training is a continuous effort. All FETPs have a regional or programme representative involved in finalizing the process for programmes to apply for accreditation. As a result of the collaborative efforts of the global network, an accreditation manual summarizing the criteria and process to become accredited was created in May 2012. This manual will be updated with information obtained from pilot site-visits to a programme in each region, so that interested programmes can apply to the process in 2014. This shows the commitment of the global network to improving quality, producing valuable outcomes and increasing the public health capacity of countries through graduates qualified to address first hand, emerging, re-emerging and endemic public health issues.

## Discussion

This article represents the first evaluation of FETP progress toward increasing public health capacity, utilizing data and information abstracted from all FETP programmes worldwide. Fifteen years ago the global network of FETPs comprised only 10 programmes, and the FETPs and graduates were recognized for ‘supporting an evidence-based public health resource that could serve communities effectively and efficientlyʼ [1]. Since then, the numbers of graduates and the breadth of public health issues they address have grown substantially. For instance, FETP residents and graduates have participated first hand in numerous cross-border and global public health investigations or emergency responses related to, for example, natural disasters, increases in non-communicable diseases in developing countries and emerging or re-emerging threats, such as pandemic influenza. Because the FETPs focus on applying epidemiology methods in real life situations, and adapt to the public health services needed, many have been sustained for a decade or more. As a result they have become increasingly embedded in and familiar with the communities they serve, and valued for the public health services they provide.

In the next 2 years as many as 800 field epidemiologists could graduate from an FETP, some also with a master’s degree in epidemiology or public health. These highly qualified graduates are a perfect resource for addressing the IHR established by the WHO, which require countries to have national resources at the local community or primary public health level to detect and respond to events involving disease or death [12]. Therefore, FETPs and their thousands of graduates are not only evidence of a resource to serve communities effectively, but also of a resource to continue to increase public health capacity and a significant human health resource for addressing emerging and established global public health challenges.

## Conclusions

The global network of FETPs has continued to grow and adapt to meet the changing needs of public health and to improve field epidemiology training to produce skilled health professionals that increase public health capacity globally. The FETPs, with service in training as a key value, are well-integrated into their national public health infrastructures, and work in collaboration with key regional and global public health partners to tackle the most challenging public health issues. The residents and graduates continue to share best practice at scientific conferences and support their countries’ public health needs during their field training and long after. As a result of the continual growth and adaptability of the programmes, the activities of the residents and graduates and continued quality improvement, it is expected that the FETPs will continue to be a significant resource for addressing global public health issues worldwide.

## Abbreviations

CDC: Centers for disease control and prevention; EIS: Epidemiology intelligence service; FETP: Field epidemiology training programme; H1N1: Swine influenza; H5N1: Avian influenza; IHR: World Health Organization International Health Regulations; MPH: Master of Public Health; MOH: Ministry of Health; SARS: Severe acute respiratory syndrome; WHO: World Health Organization.

## Competing interests

The authors declare they have no competing interests.

## Authors’ contributions

RS developed the paper concept, collected the initial data, cross-checked and analysed data, drafted the article and implemented updates to the article based on feedback from the reviewers. DH supported the final phases of data collection and confirmed data with the FETPs, and provided input into the materials to be included as part of the evaluation, and final edits and updates to the article. PK supported data collection for his programme and for all programmes via TEPHINET meetings where he was facilitator; he helped develop the final concept of the article and the final draft submitted to reviewers. All authors approved of the final manuscript.
